# What is the impact of scoliotic correction on postoperative shoulder imbalance in severe and rigid scoliosis

**DOI:** 10.1186/s12891-021-04763-y

**Published:** 2021-10-12

**Authors:** Shuo Yuan, Ning Fan, Yong Hai, Qichao Wu, Peng Du, Lei Zang

**Affiliations:** Department of Orthopedics, Beijing Chaoyang Hospital, Capital Medical University, Beijing, China

**Keywords:** Postoperative complications, Postural balance, Scoliosis, Surgery, Shoulder, Risk factors, Spinal fusion, Radiography

## Abstract

**Background:**

Although recent studies have investigated the risk factors for PSI, few studies have focused on the impact of scoliotic correction on postoperative shoulder imbalance (PSI), especially in severe and rigid scoliosis (SRS). The purpose of the study was to study the effect of scoliotic correction on PSI in SRS.

**Methods:**

The preoperative, postoperative, and minimum 2-year follow-up radiographs of 48 consecutive patients with SRS who underwent posterior spinal fusion surgery were evaluated. We regarded radiographic shoulder height (RSH) as a shoulder balance parameter and divided the patients into improved and aggravated groups of PSI from pre- to post-operation and from post-operation to last follow-up, respectively. In addition, patients were divided into nine groups based on the observed changes in PSI after surgery and at follow-up, and the correction rate ratios were calculated among the groups. Independent samples T test and Chi-squared test were performed between the improved and aggravated groups of PSI.

**Results:**

After surgery, the proximal thoracic curve (PTC) flexibility (*P* = 0.040), correction rate of the main thoracic curve (MTC) (*P* = 0.010), and Cobb angle of the lumbar curve (LC) (*P* = 0.037) were significantly higher, while the ratio of the correction rate of the PTC to the MTC (*P* = 0.042) was smaller in the aggravated group. At follow-up, the improved group had significantly larger PTC flexibility (*P* = 0.006), larger ratio of the correction rate of PTC to MTC (*P* = 0.046), a larger ratio correction rate of PTC to LC (*P* = 0.027), and a smaller correction rate of LC (*P* = 0.030). The correction rate ratios of the groups after surgery were as follows: negative to negative (N-N) (1.08) > negative to balance (N-B) (0.96) > negative to positive (N-P) (0.67), B-N (1.26) > B-B (0.94) > B-P (0.89), and P-N (0.34) > P-P (0.83). The order of the correction rate ratio at follow-up was as follows: N-N (0.96) > N-B (0.51), B-B (0.97) > B-P (0.90), and P-B (0.87) > P-P (0.84).

**Conclusion:**

Harmonizing the correction rate ratio of the PTC, MTC, and LC should be recommended for intraoperative correction and postoperative compensation of PSI. In addition, greater PTC flexibility plays an important role in the spontaneous correction and compensation of PSI in SRS.

## Background

Severe and rigid scoliosis (SRS), in which the major curve is over 80° on normal films, and the flexibility of the major curve is below 30% on bending films [1, 2], is a complex, progressive, and disabling deformity that seriously affects the patient’s quality of life. Several scoliosis correction methods have been reported for SRS and achieved satisfactory correction rates [3–6]. However, these surgical treatments remain challenging due to the high risk of perioperative and postoperative complications, such as respiratory insufficiency, neuromuscular dysfunction, cerebrospinal fluid leakage, epidural hematoma, aortic injury, and instrumental failure [7–10].

Postoperative shoulder imbalance (PSI) is one of the most notable complications and greatly affects the patient’s appearance and satisfaction [11]. Thus, surgeons should consider the incidence of PSI during orthopedic surgery, although its definition remains controversial [11]. Radiographic shoulder height (RSH) is more commonly used, and defined as the height difference between the right and left soft tissue shadows directly superior to the acromioclavicular joint on standing posteroanterior radiographs, and a difference < 1 cm is considered the upper limit of balance [12, 13]. In addition, the risk factors of PSI in adolescent idiopathic scoliosis (AIS) remain controversial, and several of these factors have been evaluated in recent years, including the level of upper instrumented vertebrae (UIV), greater percentage correction of the main thoracic curve (MTC), higher postoperative sacral slope, postoperative UIV tilt angle, and adding-on angle [14–19].

Achieving postoperative shoulder symmetry remains a challenging goal, especially for RSR. Assessing the relationship between correction surgery and PSI may further enhance our understanding of this phenomenon and could be valuable in reducing the rate of PSI. However, few studies have focused on PSI in SRS [20]. Therefore, the purpose of our study was to identify the effect of scoliotic correction on the correction and compensation of PSI in SRS.

## Methods

Of the 61 patients with RSR who were reviewed, 13 patients were excluded; thus, a retrospective review of 48 consecutive patients (15 men and 33 women) was conducted from 2008 to 2013 at a single institution. The exclusion criteria included tethered cord syndrome, neurofibromatosis, diastematomyelia, postoperative severe neurological complications, history of revision surgery or spinal osteotomy, and < 2 years of follow-up. Of the 13 excluded patients, 2 patients underwent revision surgery after spinal osteotomy, 2 patients had neurofibromatosis with postoperative paraplegia, 4 patients were excluded for diastematomyelia or tethered cord syndrome, and 5 patients for < 2 years of follow-up. Of the 48 included patients, scoliosis (all right-curve) was idiopathic in 15 patients and congenital in 33 (19 left-curve and 14 right-curve). The average age of the patients was 20.7 ± 5.4 years (range, 12–29 years). Preoperative standing full-length PA, lateral and side-bending radiographs, and postoperative and final follow-up standing full-length PA and lateral radiographs were collected. The investigation was approved by the hospital’s institutional review board, and subjects provided informed consent prior to participation. All methods in the study were carried out in accordance with the Helsinki guidelines and declaration.

### Surgical procedures

Preoperative traction, combined occipital-jaw traction, and skin traction of the lower extremity were performed on patients over the course of a week to obtain soft tissue release, improve pulmonary function, and avoid surgical complications, particularly spinal cord injury. Patients were also asked to blow balloons and climb stairs to improve their cardiopulmonary function before surgery. There were no complications during traction, and no significant improvement in scoliosis deformity was observed after traction. All operations were performed by the same senior surgeon using only posterior pedicle screw fixation. Spinal osteotomy was not routinely performed in this study. The UIV were selected based on the relative stiffness of the proximal thoracic curve (PTC) and preoperative shoulder level. The last touching vertebra (LTV) was chosen as the lower instrumented vertebra (LIV). The intraoperative surgical procedures included rod rotation, distraction on the concave side, compression on the convex side, and posterior release with facet joint resection and interspinous-supraspinous ligament resection. All surgeries were performed with intraoperative monitoring using somatosensory evoked potentials and magnetic motor evoked potentials.

### Radiographic measurements and grouping

We used the RSH (> 1 cm) to define PSI and divided the patients into balanced and imbalanced shoulder groups. Negative RSH represented ipsilateral shoulder elevation of the main curvature, whereas positive RSH represented contralateral elevation. In addition, patients with decreased absolute RSH values were classified into the improved group, and those with increased absolute RSH values were classified into the aggravated group. We then divided the patients into improved and aggravated groups of PSI from pre- to post-operation, and from post-operation to the last follow-up, respectively. Related parameters, including RSH, Cobb angles of PTC, MTC, and lumbar curve (LC), were measured; the flexibility, correction rate, and correction rate ratio were calculated; and clinical parameters, including sex, age, Risser sign, and apical vertebra were recorded.

Curve flexibility was measured by the curve magnitude on preoperative standing full-length PA and side-bending radiographs. Flexibility was calculated as follows: (preoperative standing full-length PA Cobb angle – side-bending Cobb angle) / preoperative standing full-length PA Cobb angle × 100%. The curve correction rate was determined by measuring the magnitudes of the curves on preoperative and postoperative standing full-length AP radiographs. The correction rate was calculated as follows: (preoperative standing full-length PA Cobb angle – postoperative standing full-length PA Cobb angle) / preoperative standing full-length PA Cobb angle × 100%. In addition, the correction rate ratio was calculated as follows: (correction rate of PTC + correction rate of LC) / (correction rate of MTC * 2).

The patients were divided into nine groups based on the changes in shoulder imbalance from pre- to post-operation, and from post-operation to last follow-up, respectively. The groups in which PSI changed from pre- to post-operative included the following: negative imbalanced shoulder preoperatively to negative imbalanced shoulder postoperatively (N-N), negative imbalanced shoulder preoperatively to balanced shoulder postoperatively (N-B), negative imbalanced shoulder preoperatively to positive imbalanced shoulder postoperatively (N-P), balanced shoulder preoperatively to negative imbalanced shoulder postoperatively (B-N), balanced shoulder preoperatively to balanced shoulder postoperatively (B-B), balanced shoulder preoperatively to positive imbalanced shoulder postoperatively (B-P), positive imbalanced shoulder preoperatively to negative imbalanced shoulder postoperatively (P-N), positive imbalanced shoulder preoperatively to balanced shoulder postoperatively (P-B), and positive imbalanced shoulder preoperatively to positive imbalanced shoulder postoperatively (P-P). Similarly, the groups in which PSI changed from post-operation to the last follow-up included N-N, N-B, N-P, B-N, B-B, B-P, P-N, P-B, and P-P. The correction rate and correction rate ratios were analyzed to identify the effect of scoliotic correction (PTC, MTC, and LC) on PSI.

### Statistical analyses

Variables are presented as the mean ± standard deviation. Analysis of variance (ANOVA) was used to compare the three scoliosis groups (idiopathic scoliosis, left-curve congenital scoliosis, and right-curve congenital scoliosis). Independent samples T test and Chi-squared test were performed to compare continuous variables between the improved and aggravated PSI groups. Data analysis was performed using SPSS (version 24.0; SPSS, Chicago, IL, USA). Statistical significance was set at *P* < 0.05.

## Results

**Fig. 1:**
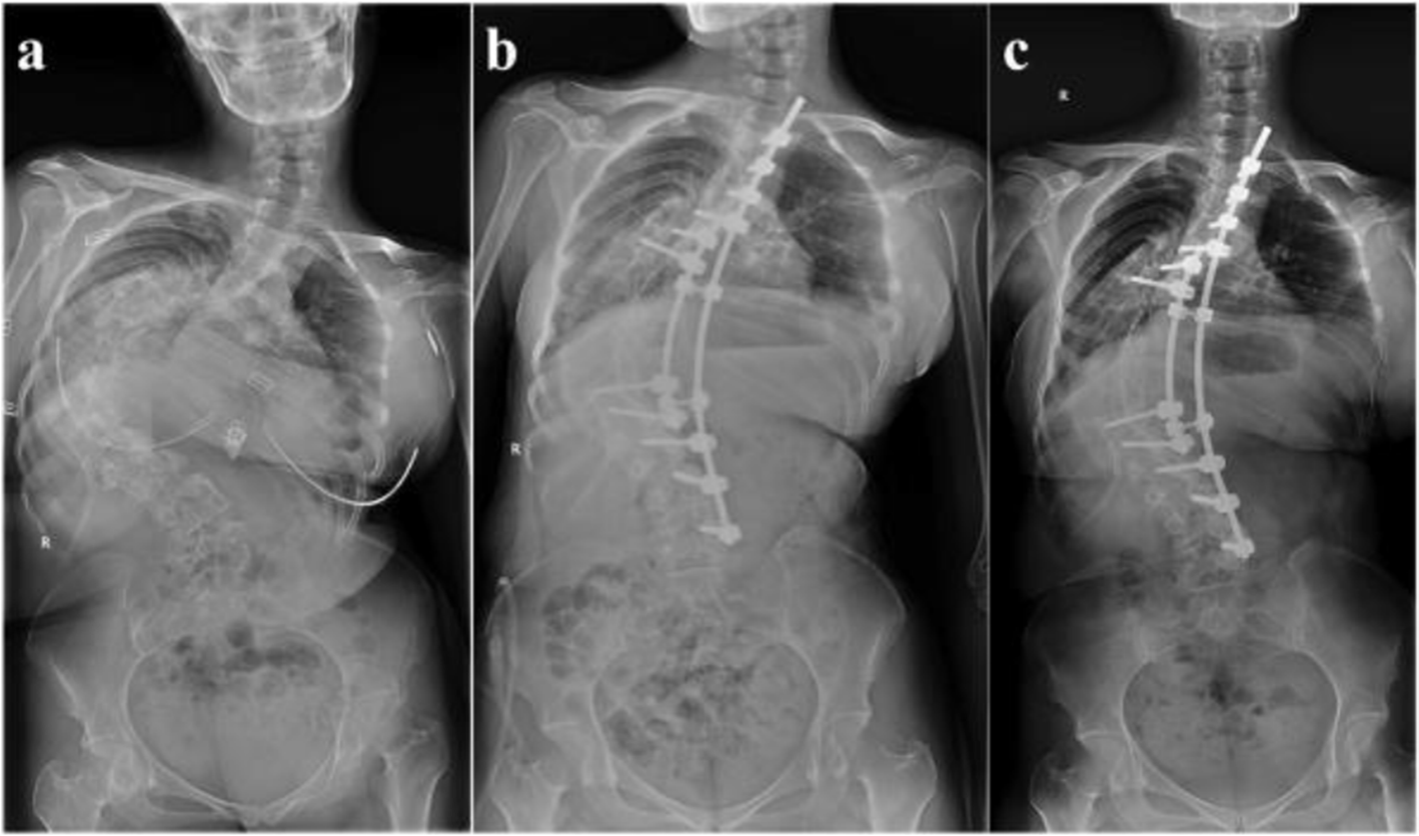
Radiographs obtained in a 30-year-old girl diagnosed idiopathic scoliosis. The patient preoperatively showed proximal, middle, and distal curvatures of 53.40°, 128.40°, and 42.70°, respectively, and RSH − 75.86 mm (**a**). Immediately after surgery, the patient showed proximal, middle, and distal curvatures of 47.00°, 85.30°, and 23.70°, respectively, and RSH − 43.71 mm (**b**). The patient showed RSH − 26.72 mm at 2 years’ follow-up (**c**)

The mean follow-up period was 34.7 months (range, 24–52 months). The RSH was − 17.37 mm ± 21.94 mm before surgery, 1.74 mm ± 22.11 mm after surgery, and 4.61 mm ± 18.27 mm at follow-up. The average Cobb angles of the PTC, MTC, and LC were 45.2° ± 21.8°, 107.4° ± 15.9°, and 40.2° ± 19.4° before surgery, respectively, and 29.4° ± 18.1°, 49.0° ± 23.5°, and 15.4° ± 14.2° after surgery, respectively, yielding correction rates of 33.8% ± 36.0, 55.1% ± 18.4, and 63.2% ± 24.0% after surgery. The mean flexibility of the PTC, MTC, and LC before surgery was 24.4% ± 17.0, 16.4% ± 10.2, and 39.7% ± 23.5%, respectively. Figure 1 show the representative case.

### Similarities of radiographic parameters among three groups with different curvature types

The 48 included patients were divided into three groups as follows: an idiopathic scoliosis group (*n* = 15), a left-curve congenital scoliosis group (*n* = 19), and right-curve congenital scoliosis (*n* = 14). RSH, preoperative flexibility, and preoperative and postoperative Cobb angles of the PTC, MTC, and LC were compared among the three groups (Table 1). Interestingly, all parameters of the different curvature types were similar preoperatively and preoperatively (*P* > 0.05), but there was a significant improvement in PSI, PTC, MTC, and LC (*P* < 0.05) after surgery.

**Table 1:** Radiographic Parameters of Idiopathic and Congenital Scoliosis

	Idiopathic scoliosis	Left-curve congenital scoliosis	Right-curve congenital scoliosis	*P*	Total
Radiographic shoulder height					
Preoperative (mm)	−20.96 ± 20.51	−13.91 ± 22.71	−18.20 ± 23.25	0.649	−17.37 ± 21.94
Postoperative (mm)	3.62 ± 22.67	−0.10 ± 16.43	2.23 ± 28.83	0.888	1.74 ± 22.11
*P*	0.004*	0.039*	0.049*	–	< 0.001*
Proximal thoracic curve					
Preoperative (°)	49.3 ± 14.2	41.0 ± 25.1	46.6 ± 24.1	0.537	45.2 ± 21.8
Postoperative (°)	30.4 ± 17.2	27.5 ± 18.2	30.9 ± 19.9	0.850	29.4 ± 18.1
*P*	0.003*	0.066	0.071	–	< 0.001*
Flexibility (%)	22.2 ± 13.5	27.1 ± 15.3	23.1 ± 22.5	0.668	24.4 ± 17.0
Main thoracic curve					
Preoperative (°)	106.7 ± 15.0	106.7 ± 16.4	109.1 ± 17.0	0.893	107.4 ± 15.9
Postoperative (°)	43.1 ± 25.2	49.9 ± 21.2	54.0 ± 24.8	0.458	49.0 ± 23.5
*P*	< 0.001*	< 0.001*	< 0.001*	–	< 0.001*
Flexibility (%)	16.9 ± 10.0	17.3 ± 9.7	14.8 ± 11.5	0.774	16.4 ± 10.2
Lumbar curve					
Preoperative (°)	41.2 ± 18.4	36.7 ± 20.7	43.9 ± 19.2	0.571	40.2 ± 19.4
Postoperative (°)	12.2 ± 8.9	13.7 ± 11.0	21.0 ± 20.7	0.200	15.4 ± 14.2
*P*	< 0.001*	< 0.001*	0.006*	–	< 0.001*
Flexibility (%)	33.0 ± 25.0	42.0 ± 25.1	43.7 ± 19.4	0.410	39.7 ± 23.5
Total	15	19	14	–	48

Negative value in radiographic shoulder height represents the ipsilateral shoulder elevation of main thoracic curve.

Positive value in radiographic shoulder height represents the contralateral shoulder elevation of main thoracic curve.

*Statistical significance: *P* < 0.05

### Comparisons of the parameters between the aggravated and improved groups after surgery

The preoperative and postoperative parameters were compared between the aggravated and improved groups from pre-operation to post-operation (Table 2). The RSH of the aggravated group (*n* = 21) was 13.37 mm ± 12.50 mm before surgery and 24.31 mm ± 13.91 mm after surgery, and the RSH of the improved group (*n* = 27) was 27.96 mm ± 18.73 mm before surgery and 12.10 mm ± 10.54 mm after surgery. The RSH was significantly larger before surgery and smaller after surgery in the improved group compared with the aggravated group. Compared with the improved group, the preoperative PTC flexibility and Cobb angle of the LC were significantly greater in the aggravated group. In addition, the aggravated group had a significantly larger MTC correction rate and a smaller ratio of the correction rate of the PTC to the MTC compared with the improved group.

**Table 2:** Comparisons of the Scoliotic Parameters in the Aggravated and Improved Shoulder Balance Groups after Surgery

	Aggravated Group (*n* = 21)	Improved Group (*n* = 27)	*P*
Age (yr)	20.52 ± 5.17	20.85 ± 5.59	0.836
Risser grade	4.29 ± 0.78	4.44 ± 0.70	0.463
Radiographic shoulder height			
Preoperative (mm)	13.37 ± 12.50	27.96 ± 18.73	0.004*
Postoperative (mm)	24.31 ± 13.91	12.10 ± 10.54	0.001*
Preoperative parameters			
Proximal thoracic curve (°)	43.51 ± 23.28	46.53 ± 20.94	0.640
Flexibility (%)	30.08 ± 17.18	19.99 ± 15.83	0.040*
Main thoracic curve (°)	105.85 ± 13.19	108.62 ± 17.85	0.555
Flexibility (%)	15.82 ± 11.45	16.90 ± 9.32	0.719
Apical vertebra			0.933
T8 or above	4	6	
T9, T10	9	12	
T11 or below	8	9	
Lumbar curve (°)	46.79 ± 19.80	35.08 ± 17.78	0.037*
Flexibility (%)	36.09 ± 24.15	42.49 ± 23.01	0.355
Postoperative parameters			
Proximal thoracic curve (°)	26.17 ± 16.34	31.89 ± 19.20	0.281
Correction rate (%)	39.12 ± 28.03	29.65 ± 41.28	0.372
Main thoracic curve (°)	46.12 ± 21.44	51.19 ± 25.14	0.464
Correction rate (%)	62.75 ± 15.71	49.17 ± 18.49	0.010*
Lumbar curve (°)	18.77 ± 16.25	12.69 ± 12.07	0.144
Correction rate (%)	59.71 ± 27.21	66.00 ± 21.27	0.373
Ratio of curve correction rate			
Proximal / Main (%)	35.78 ± 98.30	79.23 ± 39.65	0.042*
Proximal / Lumbar (%)	65.12 ± 49.57	51.82 ± 56.86	0.400
Lumbar / Main (%)	107.92 ± 45.60	128.09 ± 40.46	0.112

*Statistical significance: *P* < 0.05

### Comparisons of the parameters between the aggravated and improved groups at follow-up

Comparisons of the preoperative and postoperative parameters between the aggravated and improved groups from the postoperative to the last follow-up are shown in Table 3. The RSH of the aggravated group was 10.20 mm ± 7.36 mm after surgery and 19.92 mm ± 9.65 mm at follow-up, and the RSH of the improved group was 22.19 mm ± 14.51 mm after surgery and 12.26 mm ± 10.59 mm at follow-up. In addition, the RSH was significantly larger after surgery and smaller at follow-up in the improved group compared with the aggravated group. The PTC flexibility and the ratio of the correction rate of PTC to MTC and PTC to LC were significantly greater in the improved group. However, the aggravated group had a larger LC correction rate than the improved group.

**Table 3:** Comparisons of the Scoliotic Parameters in the Aggravated and Improved Shoulder Balance Groups at Follow-up

	Aggravated Group (*n* = 19)	Improved Group (*n* = 29)	*P*
Age (yr)	20.16 ± 5.76	21.07 ± 5.15	0.570
Risser grade	4.37 ± 0.60	4.38 ± 0.82	0.960
Radiographic shoulder height			
Preoperative (mm)	17.81 ± 11.55	24.04 ± 20.63	0.237
Postoperative (mm)	10.20 ± 7.36	22.19 ± 14.51	0.002*
Follow-up (mm)	19.92 ± 9.65	12.26 ± 10.59	0.015*
Preoperative parameters			
Proximal thoracic curve (°)	42.96 ± 21.36	46.68 ± 22.34	0.570
Flexibility (%)	16.25 ± 10.04	29.75 ± 18.63	0.006*
Main thoracic curve (°)	109.47 ± 14.48	106.06 ± 16.85	0.473
Flexibility (%)	18.28 ± 11.51	15.21 ± 9.25	0.313
Apical vertebra			0.780
T8 or above	3	7	
T9, T10	9	12	
T11 or below	7	10	
Lumbar curve (°)	44.21 ± 17.35	37.57 ± 20.49	0.250
Flexibility (%)	35.41 ± 22.90	42.50 ± 23.83	0.312
Postoperative parameters			
Proximal thoracic curve (°)	30.86 ± 18.98	28.42 ± 17.69	0.653
Correction rate (%)	23.70 ± 47.20	40.40 ± 25.20	0.117
Main thoracic curve (°)	45.04 ± 24.99	51.54 ± 22.52	0.354
Correction rate (%)	59.93 ± 19.42	51.96 ± 17.40	0.145
Lumbar curve (°)	11.29 ± 9.28	18.01 ± 16.30	0.111
Correction rate (%)	72.44 ± 19.89	57.22 ± 24.83	0.030*
Ratio of curve correction rate			
Proximal / Main (%)	34.12 ± 94.53	77.33 ± 51.47	0.046*
Proximal / Lumbar (%)	36.76 ± 55.60	71.32 ± 48.53	0.027*
Lumbar / Main (%)	126.40 ± 31.77	114.60 ± 49.71	0.364

*Statistical significance: *P* < 0.05

### Changes in shoulder balance after surgery and at follow-up

The negative imbalanced shoulder group, the balanced shoulder group, and the positive imbalanced shoulder group comprised 30, 13, and 5 patients before surgery; 15, 14, and 19 after surgery; and 12, 17, and 19 at follow-up (Table 4). The patients were divided into nine groups based on the changes in shoulder balance from pre- to post-operation and from post-operation to the last follow-up (Table 5). The correction rate of each curve and the correction rate ratio in each group after surgery were compared among the groups. The correction rate ratios after surgery were as follows: N-N (1.08) > N-B (0.96) > N-P (0.67), B-N (1.26) > B-B (0.94) > B-P (0.89), and P-N (0.34) > P-P (0.83). Similarly, the correction rate ratios at follow-up were as follows: N-N (0.96) > N-B (0.51), B-B (0.97) > B-P (0.90), and P-B (0.87) > P-P (0.84).

**Table 4:** Balanced and Imbalanced Shoulder Groups before Surgery, after Surgery and at Follow-up

	Negative Imbalanced Shoulder Group (RSH < − 10 mm)	Balanced Shoulder Group (− 10 mm ≦ RSH ≦ 10 mm)	Positive Imbalanced Shoulder Group (RSH > 10 mm)
Preoperative	30	13	5
Postoperative	15	14	19
Follow-up	12	17	19

Negative imbalanced shoulder represents the ipsilateral shoulder elevation of main thoracic curve

Positive imbalanced shoulder represents the contralateral shoulder elevation of main thoracic curve

RSH indicates radiographic shoulder height

**Table 5:** Changes of Shoulder Balance after Surgery and at Follow-up

	N-N	N-B	N-P	B-N	B-B	B-P	P-N	P-B	P-P
Changes after Surgery									
Number of patients	12	10	8	2	4	7	1	0	4
Correction rate of proximal thoracic curve (%)	30.79	34.95	15.18	42.19	41.69	46.26	44.10	–	40.62
Correction rate of main thoracic curve (%)	40.53	56.55	63.99	54.01	60.27	66.01	64.00	–	51.67
Correction rate of lumbar curve (%)	55.16	70.76	66.96	88.39	69.37	68.70	0	–	48.86
Correction rate ratio	1.08	0.96	0.67	1.26	0.94	0.89	0.34	–	0.83
Changes between after surgery and at Follow-up									
Number of patients	12	3	0	0	8	6	0	6	13
Correction rate of proximal thoracic curve (%)	28.45	37.33	–	–	42.76	48.15	–	32.10	44.95
Correction rate of main thoracic curve (%)	39.36	57.45	–	–	57.58	61.55	–	61.73	60.67
Correction rate of lumbar curve (%)	46.19	21.09	–	–	66.64	63.35	–	72.36	58.27
Correction rate ratio	0.96	0.51	–	–	0.97	0.90	–	0.87	0.84

N indicates negative imbalanced shoulder; B, balanced shoulder; P, positive imbalanced shoulder

N-B represents that the negative imbalanced shoulder preoperatively to balanced shoulder postoperatively or that the negative imbalanced shoulder postoperatively to balanced shoulder at follow-up

Correction rate ratio = (Correction rate of proximal thoracic curve + Correction rate of lumbar curve) / (Correction rate of main thoracic curve * 2)

## Discussion

In our previous studies, we analyzed the risk factors of PSI and distal adding-on in SRS [2, 20]. In the present study, we assessed the relationship between scoliotic correction and postoperative changes in PSI in SRS and found that PTC flexibility played an important role in the correction and compensation of PSI at follow-up. In addition, a larger correction rate of MTC after surgery might cause or aggravate PSI, although there was no significant difference between the aggravated and improved groups at follow-up. Furthermore, the correction rates of PTC, MTC, and LC as a whole were significantly related to PSI; thus, harmonizing the correction rate ratio of PTC, MTC, and LC should be recommended for intraoperative correction and postoperative compensation of PSI.

It is important to recognize the flexibility of PTC, which plays a decisive role in the choice of UIV [18, 21]. PTC flexibility is related to spontaneous correction and compensatory ability in PSI. Chan et al. found that Lenke 1+ (preoperative PT side bending [CSSB] Cobb angle 15°–24.9°) had a worse compensatory ability and a higher risk of PSI than Lenke 1– (preoperative PTSB Cobb angle < 15°) [13, 22]. Similarly, our study showed that PTC flexibility was significantly lower in the improved group after surgery but higher in the improved group at follow-up, indicating that higher flexibility of PTC can contribute to spontaneous correction and compensation of PSI at follow-up.

Correction of scoliotic curves is considered to play an important role in shoulder balance [23]. However, the correction of scoliotic curves for preventing PSI remains controversial. Studies performed by Chang et al. [24], Moorthy et al. [15], and Ohrt-Nissen et al. [25] showed that less correction of the MTC was needed to achieve better shoulder balance in AIS. Our results showed that the aggravated group had a significantly larger correction rate of the MTC than the improved group postoperatively, suggesting that overcorrection of MTC was associated with the aggravation of shoulder imbalance. However, there was no significant difference between the aggravated and improved groups at follow-up. We speculate that spontaneous correction in PTC and LC occurred after MTC fusion and played an important role in postoperative PSI compensation.

In addition, we found that the ratio of the correction rate of PTC to MTC was significantly larger in the improved group after surgery, the correction rate of LC was smaller, and the ratio of the correction rate of PTC to MTC and PTC to LC was larger in the improved group at follow-up. These findings indicate a significant correlation between the correction rates of the PTC, MTC, and LC and the intraoperative correction and postoperative compensation of PSI. These findings are consistent with those of previous reports. Berlin et al. suggested that a moderate correction of PTC is critical for PSI [14], while Okada et al. showed that excessive correction of the lumbar curve of > 73% increased the risk of PSI in patients with Lenke type 5C curves [26]. In addition, Lee et al. found that the postoperative PTC/MTC ratio might be an important factor in the onset of PSI [27]. Similarly, Sielatycki et al. suggested that PTC must be carefully scrutinized to optimize shoulder balance, especially when larger correction of the MTC is performed [28].

To assess the relationships between the correction rates of the PTC, MTC, and LC as a whole and the changes in PSI, we divided all patients into nine groups based on different PSI changes and calculated the correction rate ratio postoperatively. We found that excessively large or small ratios would lead to PSI after surgery and affect postoperative compensation of PSI; however, we are currently unable to determine the appropriate correction rate ratio. Therefore, harmonizing the correction rate ratio of PTC, MTC, and LC intraoperatively should be recommended for intraoperative correction and postoperative compensation of PSI. We first attempted to use the correction rate ratio to describe the effect of the correction rate of the three curves (PTC, MTC, and LC) on PSI and believe that this ratio can be used to guide orthopedic procedures intraoperatively and predict the postoperative compensation of PSI. However, further research is required in this area.

This study has several limitations. First, this is a retrospective single-center study, and the evidence was therefore not as compelling as that in prospective studies. Second, the small sample size may have led to selection bias. Third, although we used radiographs immediately after surgery (1 week after) to assess the PSI, the natural state of the shoulder may be affected when the patient walks 1–2 weeks postoperatively because the body is still adapting to the orthopedic surgery; this may result in a difference between the PSI status immediately after the surgery and the real PSI. Finally, we did not employ preoperative, postoperative, or follow-up clinical questionnaires, such as the Scoliosis Research Society-22 score, to evaluate the satisfaction of patients with their clinical outcomes. Although our review had those limitations, we believe that they do not substantially detract from the conclusions of this study.

## Conclusions

Harmonizing the correction rate ratio of the PTC, MTC, and LC should be recommended for intraoperative correction and postoperative compensation of PSI. Moreover, larger PTC flexibility also plays an important role in the spontaneous correction and compensation of PSI in SRS.

## Data Availability

The datasets used and/or analysed during the current study are available from the corresponding author on reasonable request.

## Abbreviations

PSI: Postoperative shoulder imbalance
SRS: Severe and rigid scoliosis
RSH: Radiographic shoulder height
PTC: Proximal thoracic curve
MTC: Main thoracic curve
LC: Lumbar curve
AIS: Adolescent idiopathic scoliosis
UIV: Upper instrumented vertebrae
LTV: Last touching vertebra
LIV: Lower instrumented vertebra
ANOVA: Analysis of variance
